# Supporting Advance Care Planning Among Mandarin and Cantonese Speaking Communities: A Qualitative Exploratory Study

**DOI:** 10.3390/curroncol33050288

**Published:** 2026-05-14

**Authors:** Upma Chitkara, Ashfaq Chauhan, Ramya Walsan, Mary Li, Eric Yeung, Ursula M. Sansom-Daly, Reema Harrison

**Affiliations:** 1 Centre for Health Systems and Safety Research, Australian Institute of Health Innovation, Macquarie University, North Ryde, Sydney 2109, Australia; ramya.walsan@mq.edu.au (R.W.); yanmary.li@mq.edu.au (M.L.); reema.harrison@mq.edu.au (R.H.); 2 CanRevive Incorporated, George Street, Sydney 2000, Australia; eric.yeung@canrevive.com; 3 Behavioural Sciences Unit, Discipline of Paediatrics and Child Health, School of Clinical Medicine, Medicine & Health, University of New South Wales, Randwick, Sydney 2033, Australia; ursula@unsw.edu.au; 4 Kids Cancer Centre, Sydney Children’s Hospital, Randwick, Sydney 2031, Australia; 5 Sydney Youth Cancer Service, Nelune Comprehensive Cancer Centre, Prince of Wales Hospital, Randwick, Sydney 2031, Australia

**Keywords:** advance care planning, cancer, culturally and linguistically diverse (CALD), engagement, ethnic minorities, Mandarin and Cantonese speaking backgrounds

## Abstract

Whilst advance care planning (ACP) is important to ensure person-centred end of life care, there is sparse evidence about factors contributing towards engagement for people from Mandarin and Cantonese speaking backgrounds (MCSB) affected by cancer. This study aimed to establish barriers and facilitators for quality ACP among people from MCSB with cancer and carers. A qualitative study utilising semi-structured interviews and focus groups was conducted. Participants included adult community members from MCSB in New South Wales who had accessed cancer care services in Australia as a support person or a patient in the last five years with recruitment done purposefully. Data collected from eligible consenting participants were audio/video recorded, transcribed verbatim and analysed using the Framework Method applying the Theoretical Domains Framework. Eighteen people participated (11 in two focus groups, seven individual interviews). Key barriers to engagement with ACP were unclear understanding of process and conduct, poor quality communication by healthcare staff, resource constraints and cultural misalignment of ACP concepts. The main facilitators were openness of participants to discussions, culturally informed community resources and dedicated ACP services. Co-design provides a useful approach to address varied identified factors. At the system and service level, co-design with these communities and healthcare providers could potentially develop resources to assist these communities in engaging with ACP, including preparing for ACP communication. Understanding and acknowledging cultural factors that impact ACP and integrating this knowledge in ACP communication may enhance engagement.

## Simple Summary

Advance care planning (ACP) is a way to plan future medical care according to one’s own wishes. Multicultural communities have poor engagement in ACP. Little is known about what affects engagement, especially Mandarin and Cantonese speaking people affected by cancer in Australia. We aimed to address this gap in knowledge, using interview methods. This study found that lack of knowledge about ACP and how to participate, poor quality of communication about ACP by healthcare staff, differences in cultural beliefs regarding ACP, lack of time and resources, and psychological effects of cancer led to less involvement. However, openness to discuss wishes, culturally sensitive community support/resources and dedicated ACP services enabled involvement. We recommend actively involving people from Mandarin and Cantonese backgrounds with cancer alongside healthcare staff to prepare resources that can help the community to get involved in ACP, especially ones that support better communication.

## 1. Introduction

Advance care planning (ACP) has been described as “the ability to enable individuals to define goals and preferences for future medical treatment and care, to discuss these goals and preferences with family and health-care providers, and to record and review these preferences if appropriate [1] (e546). Individuals are supported to explore and share their personal values, life goals, and preferences regarding future health care, irrespective of their current age and health status through ACP [1,2,3]. As an ongoing process, ACP is relevant to individuals accessing healthcare across the care continuum from diagnosis to end-of-life, and within palliative care. Through a person-centric approach, ACP can facilitate informed decision- making by engaging families and health care staff in the development of care of plans aligning with patients’ values, goals and personal preferences [1,2]. ACP is particularly valued for individuals with poor prognosis to exercise their autonomy in decision-making for stages of life in which they are not able to make these decisions themselves [4].

As a potentially life-limiting disease, ACP is important for people affected by cancer due to reasons including complex treatment regimen, rapid disease progression and frequent change in treatment planning [5]. Despite this, ACP remains underused in cancer care [5,6,7]. Findings from the Australian National Advance Care Directive Prevalence study (2019) suggested that only 27% of older people (≥65 years of age) with cancer had documented their future medical preferences; the finding aligns with low rates of engagement with ACP and documentation reported internationally [8,9,10,11]. An umbrella review exploring range of patient, provider and health service factors contributing to underutilisation of ACP in cancer care identified cultural and religious beliefs influencing ACP utilisation [5].

ACP is particularly important for people from culturally and linguistically diverse (CALD) backgrounds with cancer to ensure person-centred care, given the variety of perceptions of cancer and expectations of treatment [12]. This is because cultural beliefs, communication norms, and expectations of cancer care in CALD populations can differ markedly from those embedded in mainstream health systems. In the Australian context, CALD is a term used to describe people who were born overseas, speak a language other than the official national languages and/or have lower proficiency of native or national languages, and/or who have parents who were born overseas [3]. Research indicates low ACP uptake among people from CALD backgrounds when compared to the general population nationally [13]. One large prospective audit study of 4187 records of adults aged 65 years and over reported lower prevalence of completed advance care directives (ACDs) among those born overseas (21.9%) compared to those born in Australia (28.9%) [13].

Many factors may contribute to lower engagement in ACP for people from CALD backgrounds with cancer [14,15,16]. ACP being a Western concept for people from CALD backgrounds has been argued as a major factor; however, evidence exists that healthcare-related factors such as low clinician confidence to deliver individualised and culturally sensitive conversations around ACP impact engagement in the process [15,16]. The potential for stoicism affecting expression of pain, association of opioids with imminent death, taboos around discussion of death, preference for home care and place of death and use of complementary medicine are other cultural factors that have been cited as potentially impacting the meaningful engagement of CALD communities in ACP [17]. Differences in perspectives about whether and when to disclose diagnosis of cancer or prognosis may also impact engagement with ACP. Advances in medicine have transformed cancer into a chronic disease, enabling many patients to live with it for years (even if it is terminal); yet, within some CALD communities, perceptions of associating cancer with fatalism, stigma and an inevitable death sentence have been reported [17,18]. A recent qualitative study published in 2025 with 31 healthcare staff and interpreters involved in providing care to people from CALD backgrounds with cancer in Australia showed that the skills of clinicians and interpreters, staff knowledge of cultural factors, care setting and physical environment, and resources to support and conduct ACP conversations were factors impacting ACP communication [14].

People from Mandarin and Cantonese speaking backgrounds (MCSBs) are among the five largest groups of migrants speaking a community language (non-English) at home in Australia (17.3%) [19]. A large proportion of this population have low English proficiency (24–26%). Nationally, two qualitative studies conducted with older people from MCSBs identified low awareness and knowledge of ACP in this population group [20,21]. Low awareness and knowledge of ACP were identified as barriers, with cultural misalignment largely dispelled as a factor impacting engagement with ACP [20]. Findings from a recent study, conducted in 2023, demonstrated that previous involvement in significant events such as illnesses or death of loved ones, involvement in end of life decision-making and exposure to ACP facilitated participants overcoming the cultural taboo of death and articulating their values and preferences [21]. However, these studies did not include people from MCSBs affected by cancer. In Australia, a cross-sectional study with people from MCSBs identified no perceived need, a lack of knowledge and believing it was too complicated as barriers to engagement in ACP [22]. Facilitators identified were English speaking proficiency and perception of receipt of useful information. However, this study did not specify number of participants with cancer in a sample that included people with cancer and non-cancer diagnoses [22].

Internationally, another study focusing on members of the Chinese diaspora with cancer was conducted in the United States of America (US) [23]. It found that engagement with ACP was facilitated by respect and trust for authority and preference for clinicians to initiate ACP. Barriers included unfamiliarity and lack of urgency with ACP, belief that the future is unpredictable and uncontrollable, a desire to maintain hope through uncertainties and a sense of otherness. Evidence from Australia highlights that engagement with ACP among people from MCSBs is low; however, there is sparse evidence of factors impacting engagement among these communities in cancer care in Australia [20,22,24]. This piece of research aims to fill in this gap. The aim of this study was to explore and identify barriers and facilitators to engagement with and/or uptake of ACP among people from MCSBs with a diagnosis of cancer in Australia.

## 2. Methods and Materials

### 2.1. Design

A qualitative exploratory study comprising interviews and focus groups with health consumers with experience of cancer and consumer representatives from MCSB were used to address the aim. In the Australian context, “health consumers” are people who use, have used, or are potential users of health services [25]. Semi-structured interviews and FGs provided a flexible approach to data collection to obtain in-depth data on experiences and expectations of ACP [26,27]. In consultation with a community organisational partner and given the sensitivity of the topic of conversation, the choice of either participating in FG or a semi-structured interview was offered. The Consolidated Criteria for Reporting Qualitative Studies (COREQ) were used to report this study [28]. (Supplementary File S1).

### 2.2. Setting

This study was conducted in the state of New South Wales in Australia. According to the 2021 census, New South Wales has a total population of 8,060,511, of which, almost one-third (=32.3%) speak a language other than English at home [29]. Among them, Mandarin speaking people form the largest cohort with the Cantonese speaking group claiming the third spot, collectively forming 419,426 individuals (=16%) residing in the state. NSW also exhibits high numbers of cancer diagnosis among people from MCSBs among all diverse groups [29]. Among people diagnosed with cancer in New South Wales, 17% speak a language other than English at home. Of these, people from MCSBs rank among first five, collectively making up 2.6% of total cancer-diagnoses in the state [29].

### 2.3. Resources/Documents/Materials

Participant-facing research materials and an interview topic guide were developed in collaboration with three consumers from CALD backgrounds with lived experience of cancer. These three consumers were members of the project-steering group associated with this research project. These three consumer members also supported the research team in developing a recruitment strategy for recruiting people from Mandarin/Cantonese speaking backgrounds. This was done to facilitate purposeful recruitment and participant engagement through culturally informed recruitment strategy.

The interview guide was informed by Theoretical Domains Framework to elicit responses from the participants about a comprehensive range of barriers and facilitators that may impact engagement with ACP among people from MCSBs [30]. The topic guide first asked participant/s to introduce themselves and their background. Following this, the topic guide explored (a) experiences of ACP within Australia, including usage of resources; (b) cultural competence of healthcare system around ACP; and (c) expectations around ACP. We also sought feedback from consumer organisations and advocates on framing of questions. The topic guide was pilot-tested with a bicultural bilingual researcher ML prior to data collection and updated accordingly to ensure its clarity and ease of understanding for the target communities. One topic-guide was used for both focus groups and individual interview and was updated to include carer-specific items in line with emerging findings (Supplementary Files S2 and S3).

### 2.4. Recruitment and Eligibility

People from Mandarin or Cantonese speaking backgrounds were eligible to participate in the study if they (a) were 18 years or older, (b) self-identified as Mandarin or Cantonese speaking, (c) had accessed cancer care services in Australia as a support person or a patient in the last five years, or had been associated with/member of a group who support and advocate for consumer engagement in healthcare, palliative care and cancer care, and (d) were willing to provide consent to participate. People who were acutely ill and/or were unable to provide consent were excluded from the study.

Purposive sampling was done using a multipronged approach to data collection. Recruitment was conducted through members of the project steering committee, community networks/organisations and community leaders (ML) who facilitated the process by sharing the recruitment flyers with the contact details of the research team and/or verbally introducing the study to community members and requesting interest. ML is a Mandarin speaking research team member and a Chinese community-leader. Organisations supporting health consumers, people with cancer or their carers, CALD communities and/or specifically people from MCSB in the state of New South Wales were also reached online or in-person. Given the sensitive nature of the topic, consultation was also done by researcher UC through community engagement for strategies to approach and recruit health consumers with experience of cancer and consumer representatives. Can-Revive, a Chinese community organisation supporting people with cancer and their families was engaged to provide guidance and a platform to recruit participants. Flyers were also shared on social media by the research team. Interested participants reached out to the research team. Once potential participants contacted the research team, eligibility was confirmed. Researcher UC provided participants with information on her background and the purpose of doing this research, which facilitated development of rapport between them. Eligible participants were then provided with study information sheet and consent form (PICF) prior to data collection.

### 2.5. Identifying and Responding to Preferences and Support Needs

Language support needs of participants were identified by researcher UC after confirming eligibility. Participants requiring language support during screening and recruitment had access to a bilingual researcher to convey the information and obtain consent. Preferences for mode of data collection (focus group vs. interview and online/phone/in-person as mode of data collection) were collected from all eligible participants. If a participant preferred an individual interview, technical support was provided via one-to-one communication to facilitate online participation. Where eligible participants were unable or did not wish to use online tools, in-person individual interviews were scheduled. Transport support for travel to and from venue where data collection occurred in person and interpreters for language support during FGs and individual interviews were arranged if required.

### 2.6. Data Collection

Data collection commenced after consent was obtained and recorded. Interviews and focus-groups were conducted by researcher UC (MBBS, MPH) as an MPhil candidate. UC belongs to CALD group, has a medical background (clinician) with experience in conducting qualitative data collection with diverse health-consumers in Australia.

Participants were welcomed and introduced to person providing language support as needed, participants were briefed about the study and conduct of interviews/focus-groups. The researcher acknowledged the sensitivity of the study-topic and advised participants to inform if/when they feel any discomfort. UC facilitated discussion using the topic guide to steer the discussion but to also explore interesting lines of inquiry and enable free expression of views [31]. Participants were asked to discuss the facilitators and barriers experienced when communicating about ACP wishes and preferences. Comments, thoughts and perspectives of individuals were explored rather than looking for a consensus view [31,32]. Language support was provided through bilingual fieldworker (ML) with 15 years of research experience or a National Accreditation Authority for Translators and Interpreters (NAATI)-certified professional interpreter [31]. The content of the dialogue was audio recorded with participant consent and transcribed verbatim. Upon completion of individual interviews and focus groups, participants were provided with an option for a check-in phone call from researcher in 24 h’ time to inquire on well-being.

Data collection through in-person interviews or focus groups occurred at community centres or organisations where participants frequented thus providing them a safe and familiar environment. This approach allowed participants to feel comfortable and share their thoughts and experiences on the topic. Participants of individual interviews and focus groups were reassured about their privacy. Each focus group lasted 75–90 min while interviews lasted 30–35 min.

The sample size was initially approximated (*n* = 15), and adequacy was evaluated continuously during the research process based on emerging findings utilising principles of information-power [33]. Emerging findings from the data were discussed with the core research team (AC, RH, RW, USD) in regular fortnightly meetings, in which the need to collect more or cease data collection was also determined. Data collection was ceased when it was determined through team discussion that new knowledge had been acquired to address the study objectives method utilising information-power principles [33]. Consumers and consumer representatives were remunerated for their time to take part in the study based on the rates proposed by Health Consumers NSW [34].

### 2.7. Data Analysis

Data was analysed inductively and deductively using the Framework Method grounded in the Theoretical Domains Framework [30]. This framework was developed to synthesise 33 theories of behaviour and provided a theory-informed basis to identify barriers and enablers. Study data was managed using Microsoft excel and analyses were conducted on the software. Data analysis drew out common experiences and perceptions regarding the barriers and facilitators of engagement amongst people from MCSB experiencing cancer. At first, data were inductively coded into barriers and facilitators for engagement with ACP among people from MCSBs with cancer diagnosis. Next, barriers and facilitators were grouped into categories, followed by deductive mapping against the Theoretical Domains Framework domains which resulted in formation of themes (Table 1).

The Framework Analysis method comprising 5 steps. In step 1, three researchers (UC, AC, RW) familiarised themselves with the data by re-reading the transcripts several times. In step 2, three transcripts were independently coded inductively by the two researchers (UC, AC). The third researcher (RW) inductively coded the separate three transcripts. The three researchers got together to compare their findings (Table 1, column 1) and develop a working analytic framework. These three researchers then individually completed the coding of the remaining transcripts. During this process, they assembled fortnightly to discuss their findings and revise the working analytic framework. Additionally, findings were also deliberated over with the project lead (RH) in separate fortnightly meetings and the framework refined accordingly. Step 3 consisted of framework matrix charting by mapping of inductive codes against TDF domains on an excel sheet (Table 1, column 2). In step 4 preliminary deductive themes were developed from the above mapping. In step 5, preliminary themes underwent further refinement through discussion between research team members, adding to study rigour. Final themes were developed as shown in Table 1, column 3. Disagreements during all these steps were handled through discussions between the three researchers (UC/AC/RW) followed by a review with the project-lead (RH). Decisions were based on consensus. The drafted manuscript was reviewed by a study participant who helped with recruitment through Can-Revive and co-authored the study.

The interpretation of results might have been affected by the authors’ positionality of belonging to the CALD community. The bias was minimised through three researchers contributing to the process, and the subsequent review with the project-lead. This led to final interpretations being based on consensus after resolving any conflicts.

## 3. Results

### 3.1. Participant Characteristics (Table 2)

One participant dropped out before commencement of data collection for unknown reasons. Of the 18 participants, most were women (*n* = 13, 72%) with their ages ranging from 65 to 81 years for most. The majority of the 18 participants were individuals with a cancer diagnosis (*n* = 11, 61%), followed by carers (*n* = 6, 33%) and consumer representatives (*n* = 2, 11%). One consumer representative was a carer as well. The majority of the participants were Mandarin-speaking (*n* = 13, 72%), the rest were Cantonese speaking (*n* = 5, 27%).

Two focus groups (five participants in one focus group and six in another) and seven individual semi-structured interviews were conducted. Both focus groups were held in-person, occurring at community centres. Five interviews were conducted over the phone, two in-person and one online over MS Teams. Language support via interpreter or bilingual fieldworker was provided to all but two participants, who were proficient in English. Participant characteristics are shown in Table 2.

**Table 2:** Participant characteristics.

Participant Code	Age (Years)	Gender	Language Background	Length of Stay in Australia (Years)	Patient/Carer/CR	Cancer Type
Cr1_Int	72	M	Mandarin	>55	Carer, Consumer Rep	Lung Cancer
Pt1_Int	22	F	Cantonese	22	Patient	Blood cancer
Pt1_FG1	73	F	Mandarin	13	Patient	Breast cancer
Pt2_FG1	73	F	Mandarin	12	Patient	Breast cancer
Pt3_FG1	66	F	Mandarin	9	Patient	Lung cancer
Pt4_FG1	81	F	Mandarin	13	Patient	Lymphoma
Cr1_FG1	83	M	Mandarin	13	Carer	NA
CR_FG1	69	F	Mandarin	20	Consumer Rep	NA
Cr2_Int	Na	F	Mandarin	36	Carer	NA
Pt2_Int	71	F	Mandarin	35	Patient	Ovarian cancer
Cr3_Int	65	F	Mandarin	35	Carer	NA
Cr4_Int	65	M	Mandarin	34	Carer	NA
Pt3_Int	69	F	Cantonese	6	Patient	Lung cancer, Lymphoma
Pt1_FG2	69	F	Cantonese	35	Patient	Breast cancer
Pt2_FG2	73	M	Cantonese	27	Patient	Oesophageal-l cancer, Lung Cancer
Cr1_FG2	74	F	Cantonese	27	Carer	NA
Pt3_FG2	80	F	Mandarin	42	Patient	Breast cancer, Lymphoma
Pt4_FG2	69	M	Mandarin	2	Patient	Prostate cancer

Pt = patient; Cr = carer; CR = consumer representative; Int = Interview; FG = focus group; NA = not applicable; Na = not available.

### 3.2. Barriers and Facilitators to ACP Uptake Among People from Mandarin and Cantonese Speaking Backgrounds with Cancer

Analysis of qualitative interview data revealed a range of factors as barriers or facilitators across the domains of the Theoretical Domains Framework [30]. The factors are reported in four themes (Table 1): (1) knowledge and skills of ACP conduct; (2) resources for ACP discussion (3) cultural alignment of ACP, and (4) psychological impacts of cancer. Collectively, findings reveal that participants characterised ACP as a concept that was misaligned with their cultural values. Additionally, there was a lack of knowledge on how to engage with ACP as a process. Participants also perceived that healthcare staff had limited time, resources and skills to encourage their participation in ACP. Psychological impact due to diagnosis of cancer or illness journey further impacted their engagement in ACP.

#### 3.2.1. Theme 1: Knowledge and Skills of ACP Conduct

Engagement in ACP was dependent on participants’ knowledge of and skills in ACP communication. Participants overwhelmingly verbalised clear views about the type of care they would like to receive at the end of life. This was identified as a facilitator to engaging in ACP; however, they were unaware of the process and components of ACP through which these wishes could be expressed and had limited knowledge on how to execute it. Additionally, they also perceived that cancer service staff either did not initiate any ACP conversations or provided poor-quality communication. These two factors were identified as barriers to engagement with ACP.

When expressing the type of care they would like to receive at the end of life, some participants mentioned that they want to avoid over-treatment at the end of life and prioritise good quality of life. Desire to express these care preferences was due to awareness that a lack of ACP knowledge and skills for engagement can be detrimental in the case of a change in cancer trajectory, awareness of cancer trajectory, existing knowledge of painful resuscitation procedures and belief that best quality treatment was being provided in Australia.

*“I have already spoken to my daughter, that I do not want any over treatment at the end of my life. Do not try to resuscitate me, let me die with dignity. I do not want all the tubes on me. I want to die peacefully”.*

(P1_FG1)

*“Although I felt that breast cancer is a treatable cancer, but you never know when it will come back again”.*

(Pt2_FG1)

*“Just talking about resuscitation…has been very painful. The process of resuscitation and also that even though you have done that, there’s a chance that you might not survive as well.”*

(Pt3_FG2)

*“Why it has not talked, if you can afford to get treatment, that is already the best, no one can have extravagant hope, therefore, although they are cancer patient in Australia, they all in good spirit they are very grateful to the health system and to the Australia government.*

(CR_FG1)

Participants expressed that they were unaware of the process and components of ACP, which was identified as a barrier to engagement. Participants reported that they did not know if it was similar to writing a will, donating their bodies and funeral planning. One participant expressed frustration over not knowing how to engage with ACP. Participants were unsure if ACP documentation was legal or if the public health system provided ACP services.

*“Is this include the end of life will?… I have a different view on End-of-life care: Yesterday, I attended a talk from Cancer Council, they mentioned about a clinical trial, I would donate my body to the research.”*

(Pt4_FG1)

*“Is the similarity (of ACP) about planning for funeral?”*

(Pt2_FG2)

*“Yes, because we don’t know how to (engage with ACP)?”*

(Pt2_FG2)

*“So, the question is about whether the legal document is?”*

(Pt2_FG2)

*“We never thought we can get this kind of help from government or from anybody else.”*

(CR_FG1)

Various participants expressed that cancer services staff provided poor quality of communication about ACP, which was a barrier to their participation in these conversations. Participants highlighted that healthcare staff never initiated conversations about ACP. They noted that the conversations during clinicians’ appointments and/or chemotherapy sessions were restricted to cancer treatment only. Some participants perceived that their young age and/or good prognosis from cancer treatment might have prevented initiation of ACP conversations by healthcare staff.

*“Never, no one has ever mentioned anything about this (ACP)to me.”*

(Pt1_FG1)

*“I don’t think so, only the nurse when I first time went for chemo, she tell me what is side infection, what things can happen and what you need to do, anything related to the chemo, nothing else”.*

(Pt2_Int)

*“Actually, it wasn’t… it may be a couple of times I thought maybe it could… maybe my age was a factor… I was 18–19 when I was diagnosed and going through treatment so never received any end-of-life care or planning… because I went through stem cell transplant…the curative kind of approach…maybe that was another factor as well”*

(Pt1_Int)

*“…up to right now because everything goes to as planned. The treatment of those really on track so well, we haven’t got to that stage to discuss. I think if things go wrong, we might point to that stage to talk about those kinds of issues”*

(Cr4_Int)

Poor quality of communication about poor prognosis was a major barrier to engagement in the process. Cancer patients were communicated to about the need for ACP only in the context of a poor prognosis very close to anticipated death. Patients and families struggled to accept the prognosis, and it gave them a feeling of abandonment by healthcare team. Communication was perceived to be insensitive.

*“I don’t know if that’s true or not, but it seems to me if the communication come across as being too professional and cold, right”*

(CR1_Int)

*“So that process needs to start not when the patients about to die in two weeks’ time. It’s got to start early in the piece, and the medical care team would have some knowledge about that…, families or some family member do not understand or do not want to understand or do not accept the fact that that patients gonna die in a short period of time. There’s a lot of gap; there’s a lot of our fear…the patient have been abandoned by his treating doctors by his original medical team*

(Cr1_Int)

#### 3.2.2. Theme 2: Resources for ACP Discussion

Resources, including staff time (duration of the appointment) and provision of relevant media resources (print/online) available to facilitate ACP discussions were described as critical. Participants reported that inadequate duration of specialist appointments and lack of ACP resources were a barrier to discussions, while dedicated ACP services were potential facilitators. They verbalised that specialist appointments were short, and they were not actively provided with any ACP resources or links that could be accessed. They expressed that dedicated ACP services or a channel that can be accessed at their own convenient time may facilitate engagement in ACP.

*“I think at that stage me and doctor actually going to talk about cure as my condition was life threatening. Doctor actually talk to me, try to explain to me what’s the treatment and what is side infection, what is the schedule. Normally we see doctor for half an hour and in half hour they provide this type of information to you”*

(Pt2_Int)

*“No (I didn’t receive any resources)… I have seen a pamphlet in the dental hospital about advance care planning, but I didn’t take it”*

(Pt4_FG2)

*“So we yeah, we’re like a professional person, but we should approach them, because our time… so that we can make sure we can approach them… because lot of time spent in appointments and check-ups so…so instead of someone coming to approach us—we should be able to approach them.*

(Pt_FG2)

*“It will be good if we can have a contact person or a helpline that we can contact or to ask questions, that would be a channel for us to organise the end-of-life care.”*

(CR_FG1)

#### 3.2.3. Theme 3: Cultural Alignment of ACP

Engagement with ACP was considered to be influenced by the cultural alignment of the concept of ACP with participants from MCSBs. Participants expressed that the concept of ACP did not align with their cultural beliefs thereby acting as a barrier. For example, strong beliefs for recovery may mean that starting ACP means losing hope for recovery, asking for help with ACP meant loss of resilience.

*“It’s very difficult for the patients to be asked those kinds of questions because basically you are telling them that’s well that’s the end of the road. You have to decide what needs to do be done next. Well, initially you get a very, well… you can either get a very, well, upset person”*

(CR_Int)

*“I’m that person… very strong, yeah. I’m fine”.*

(Cr4_Int)

Participants also identified their cultural belief around family being the primary unit for ACP decision-making affecting their engagement with ACP. Participants noted a person with cancer may nominate one family member to make decisions on their behalf and this needs to be considered when conducting ACP communication. One participant also mentioned that some family members may guard the cancer diagnosis and prognosis from patient. A participant described that their community believed family was responsible for managing care towards end of life; thus, they avoided ACP conversations outside of family circle.

*“the Chinese community in particular the family unit, that’s affected by cancer, not just the patients They patient will tend to abrogate their decision-making to whoever they think they can trust… the families or some family member do not understand or do not want to understand or do not accept the fact that patients gonna die”*

(CR_Int)

*“I was shocked to hear when one of my family approached my grandmothers ‘diagnosis of cancer, my grandmother was diagnosed with… liver cancer but I found out that all of my aunts and uncles decided to not tell her, they didn’t want her to spend time worrying about her diagnosis.”*

(Pt1_Int)

*“No one wants to mention about it, we belief this is something for the family to think and to organise, it is our own responsibility.”*

(P2_FG1)

Some participants expressed that they discussed ACP in a different way among the family members, which was a facilitator of planning care towards the end-of-life within families. It was more about having a good quality of life within families.

*“It’s not that we don’t want to talk about it. It’s because we already know… we’re already 70 years… we are plan is if you can easily eat, if you can sleep, you sleep, you can treatment, do you treatment if you have to that’s it. As long as he is not suffering…”*

(Cr 3_Int)

*“Now the plan is not to talk about end of life, it is about how to live better.”*

(P3_FG1)

Numerous participants expressed that culturally informed resources and peer-support from a community cancer organisation were potentially helpful in facilitating their engagement with ACP. A participant identified a community organisation for peer-support to access guidance for engaging with ACP. A representative from one community organisation shared that community support-groups and their own involvement as a community-leader facilitated engagement with ACP.

*“(name of organisation) they have the volunteers. They asked us if you want to volunteer to help people, now they got the similar problem you got, so that it’s quite good methodology to help the people”.*

(Pt2_Int)

*“when you get a group of people together and you start easing them and talking to them about those issues, right, then it can come across bit easier”*

(CR_Int)

*“I’ve been involved in quite a lot of cases where basically I’ve been asked to assist family whose loved ones are at the end of their life, And I guess so people need a lot of help in trying to make (decisions).”*

(CR_Int)

#### 3.2.4. Theme 4: Psychological Impact of Cancer

Engagement with ACP was adversely impacted by the psychological impacts of the cancer diagnosis and care trajectory for participants at various points during illness, thereby proving to be a barrier. A participant expressed that she was overwhelmed when her cancer was diagnosed, following that she underwent various treatment modalities in quick succession which did not allow her to think about anything else. Another participant verbalised that he was emotionally burdened and struggled to engage in ACP. A representative from a community organisation shared that consumers heard and interpreted information in their own way when faced with life-or-death situations. This was perceived as a barrier to meaningful engagement with ACP.

*“I’m not even thinking about, because by that time probably you are so impact by… why it was me?… we always think cancer is someone else’s problem, never think about yourself. Where is this coming, and so many things you need to determine and treatment is one by one,…after surgery you’re not recovered then the chemo, so you’re struggling with all the new areas you need to face, you need to handle, you haven’t got enough time to think anything more!”*

(Pt2_Int)

*“….and eventually the hardest decision to make was that I decided to take her out of the hospital system and took her home but knowing by doing that I have shortened her life span. And I knew that she will die within a couple of months or even not even couple months, few weeks, rather than no stay in hospital.”*

(Cr1_Int)

*“so when people are faced with life and death and very serious situation, people tend to try to hear what they want to hear rather than what, rather than they make their own interpretations”*

(CR_Int)

## 4. Discussion

### 4.1. Principal Findings

Utilising the Theoretical Domains Framework, the study identified a range of barriers and facilitators that influenced uptake of ACP for people from MCSBs with cancer in New South Wales. Barriers and facilitators spanned from preparation of ACP communication to sharing or documenting wishes and preferences for care. Key barriers were related to misalignment between ACP concepts and cultural beliefs, unclear understanding of ACP process and conduct, poor quality ACP communication by cancer service staff, lack of resources for ACP conversations and psychological impacts of a cancer diagnosis. With sparse relevant literature in Australia, this study provides strong evidence to understand barriers and facilitators among people from MCSBs with cancer in Australia.

### 4.2. Comparison with Prior Work

Cultural misalignment of ACP acting as a barrier was a major finding of our study and aligns with prior work conducted in the US. The study identified cultural beliefs such as physicians being authority figures and expected to initiate ACP discussions, maintaining hope, and the future being unpredictable and uncontrollable as barriers among Chinese-American consumers with cancer [26]. Our study adds to above international evidence in Western settings by identifying additional cultural barriers like beliefs around hope and resilience, and family as unit in ACP decision-making with ACP discussions constrained by variation in acceptance of poor prognosis by different family members. Within Australia, cultural misalignment as a barrier aligns with another study conducted in the state of Victoria with people from MCSB in which barriers like social stigma and culturally insensitive terms used by health care staff were identified [22]. However, this study did not mention the number of participants with cancer diagnosis in a sample that also included non-cancer diagnoses. Our findings of family as a unit in decision-making as barrier also aligns with a recent qualitative study done nationally with healthcare staff which highlighted that the cultural aspect of the whole family/multiple family members being involved in ACP decision-making process added complexity to the process of ACP [14].

The present study identified that people from MCSB with cancer were open to ACP conversations due to previous exposure to or knowledge of intensive resuscitative procedures done with their loved ones. Recent evidence from a non-cancer cohort in NSW aligns with the finding that, despite lack of knowledge of ACP, previous experiences of decisional conflicts and exposure to traumatic/sudden death of loved ones acted as a facilitator to engagement [21]. A gradual cultural shift towards more open conversation around death and dying has been identified as a facilitator in Eastern settings [35]. This gradual shift may indicate individual variation in being open to ACP discussions. This highlights the importance of adopting person-centred ACP practices.

International evidence exists where healthcare providers’ paucity of cultural nuances and skills in communicating with CALD communities was identified as a barrier to engagement with ACP [36,37]. Our study aligns with this finding among people from MCSB with cancer where they found ACP communication to be of poor quality (perceived as cold and insensitive and initiated very late/near death, or lack of initiation of ACP conversation and/or provision of relevant information resources). The finding aligns with the outcomes of a national study conducted with healthcare staff and interpreters managing people from CALD backgrounds with cancer where they identified skills to conduct ACP, knowledge of cultural aspects and availability of supporting Australian resources as factors impacting implementation of ACP [14]. With both consumer- and healthcare provider-related communication barriers identified in Australia, there is a need to understand and acknowledge cultural factors that affect ACP and integrate this knowledge in ACP communication.

The finding of no or late initiation of ACP conversations from healthcare providers despite cancer diagnosis among participants was noteworthy. International studies have identified preference for ACP conversations early in disease course with consumers wanting them before diagnosis and optimal timing varying by country, considering medical care system, cancer screening programmes, and cultural factors [38,39]. An Australian cross-sectional study with 705 participants with cancer and support people (25% from CALD backgrounds) reported a preference for initiation of ACP when cancer becomes untreatable (38%) followed by at a time of their choice (30%) and at the time of diagnosis (20%) [40]. Our findings indicate that late initiation of ACP may lead to feelings of abandonment, impacting engagement with ACP when initiated late.

### 4.3. Implications

These findings indicate that barriers and facilitators operate across individual, cultural, and service levels, and cannot be addressed through single, standardised intervention. An approach is required which can fill the identified gaps around awareness and conduct of ACP, cultural alignment, psychological impacts of cancer, quality of communication of healthcare staff and availability of resources. Co-design provides a useful approach to co-create solutions with communities and healthcare providers to improve engagement in ACP [41]. Co-design intervention with the MCSB community involving the development and trial of a Chinese language ACP toolkit in the state of Victoria was successful in exploring perceived needs, barriers to ACP uptake, knowledge gaps, and perceived changes required to current practice [22]. Two-thirds of participants agreed they received useful information, which was associated with positive motivation to engage with ACP within 6 months, demonstrating a grade of success. A resource developed with communities will enhance its relevance and acceptance for use. A pilot study evaluating the feasibility and acceptability can provide the necessary evidence and information for scaling the co-designed intervention. Co-design should address the different needs and priorities of people with cancer with regard to ACP as compared to non-cancer ones [42,43]. ACP in cancer care often places a greater emphasis on clarifying end-of-life preferences, goals of care, and desired quality of life. Participants from rural settings should be included in co-design.

Evidence-based guidelines in Australia recommend ACP to be initiated early while a person still has decision-making capacity and deems it extremely important in certain scenarios including old age, chronic illness, multiple diseases and for specific religious, cultural or values-based wishes and preferences about healthcare [44,45,46]. Optimal Care Pathways for cancer emphasise that ACP should ideally start at the time of cancer diagnosis and continue through the entire sickness journey [47]. Earlier initiation may also overcome the impact of psychological distress and feelings of being overwhelmed in the context of adjustment to a person’s disease, by enabling more time for slower, patient/family-centred introductions of ACP concepts. In the context of the findings for late or no initiation of ACP leading to poor engagement in the process, ensuring implementation of Australian guidelines for recommended timing for initiation of ACP on a service-level is recommended. This time should be revisited during co-designing intervention with people from MCSB with cancer to address cultural requirements.

### 4.4. Strength and Limitations

This was the first study that the authors are aware of to provide strong evidence of factors affecting engagement with ACP among people from MCSBs with cancer from their own perspectives in Australia. This study provided flexibility in data collection methods (interviews vs. focus-groups) to facilitate participation of the CALD community in the study. In-language data collection overcame language barriers thus further supporting research participation of the CALD/MCSB community. Most of the participants were from city and its immediate suburbs. All participants belonged to New South Wales. There is a possibility of missing out on additional factors in rural dwellers and residents of other states and territories from these communities [8]. Older adults (>65 years) were overrepresented in the sample. Prior literature highlights the unique developmental needs of young adults in palliative and end-of-life-related communication [48]—this is an additional layer of complexity which our data here may not address. Use of Theoretical Domains Framework allowed in-depth exploration of factors affecting engagement with ACP. However, this may have led to the exclusion of other factors that may not fall within the domains. Findings from this study are limited to people from MCSB and do not extend to other communities. Healthcare professionals’ views were not included in this research study. However, it formed the focus of dedicated complementary study with healthcare staff and interpreters who work with people from CALD backgrounds with cancer that has been carried out within the wider programme. Voluntary Assisted Dying was not explored in this study.

## 5. Conclusions

There are a range of factors impacting engagement of people from Mandarin and Cantonese speaking backgrounds with cancer in New South Wales. At system and service levels, co-design with these communities and healthcare providers could be employed to develop resources that would assist these communities in engaging with ACP, including preparing for ACP communication. Understanding and acknowledging cultural factors that impact ACP and integrating this knowledge in ACP communication may enhance engagement.

## Figures and Tables

**Table 1:** Barriers and facilitators across Theoretical Domains Framework domains.

Inductive Coding	Mapping Against Domains of TDF	Deductive Coding
Barrier: Unclear understanding of ACP process and conduct Barrier: Poor quality ACP communication by cancer service staff	Knowledge Skills	Knowledge and skills of ACP conduct
Facilitator: Openness to discuss wishes and care preferences	Knowledge Intentions	
Barrier: Time and resource constraints to ACP conversations	Environmental Context and Resources Memory, Attention and Decision Processes	Resources for ACP discussion
Facilitator: Dedicated ACP services	Environmental Context and Resources	
Facilitator: Culturally informed community resources Facilitator: Framing of ACP within families	Environmental Context and Resources Social influences	Cultural alignment of ACP
Barrier: Misalignment between ACP concepts and Cultural beliefs	Social Role and Identity Social Influences Beliefs about consequences	
Barrier: Psychological impacts of cancer diagnosis	Emotion Beliefs about Consequences Memory, Attention and Decision Processes	Psychological Impact of cancer

## Data Availability

The data used in this study is stored and anonymized. The data is not publicly available, but it may be available upon formal request (please contact the corresponding authors).

## Supplementary Materials

The following supporting information can be downloaded at: https://www.mdpi.com/article/10.3390/curroncol33050288/s1, File S1: COREQ (Consolidated Criteria for Reporting Qualitative Studies(COREQ): 32-item checklists; File S2: Interview Guide 1: Patients/Consumer Representatives; File S3: Interview Guide 2: Carers.

## Abbreviations

The following abbreviations are used in this manuscript:

ACP	advance care planning
ACD	advance care directive
COREQ	Consolidated Criteria for Reporting Qualitative Studies
CALD	culturally and linguistically diverse
MCSB	Mandarin and Cantonese speaking backgrounds
