# Supplementary material for: Supporting Advance Care Planning Among Mandarin and Cantonese Speaking Communities: A Qualitative Exploratory Study
Source: Curr Oncol. 2026 May 14;33(5):288. doi: 10.3390/curroncol33050288 (PMC13204915; doi:10.3390/curroncol33050288)
Supplement: Supplementary file 1 [file curroncol-33-00288-s001.zip › SuppFile_02_IG__ConConReps_final_2023-10-18.pdf]

<u>Interview Guide:1</u>

<u>Topic areas</u>

1.Introductions

- Could you please tell me a bit about your background and the cancer services you have attended?

2. Knowledge, skills, capabilities and resources

- Can you tell me a bit about your experiences of communication with health professionals about EOLC planning – wishes, preferences or questions you might have had?

    - Probe – how did these conversations come about, at what stage and in what setting
    - Probe – what made it easier for you and your loved ones to talk about your wishes preferences and questions
    - Probe – what challenges did you encounter in having these conversations? did any of these challenges relate to your cultural or linguistic background?

    - To what extent do you feel that cancer service staff are equipped with the cultural knowledge and skills to approach EOLC planning with your community?
        - Probe – what do you think is lacking in skills and knowledge?
    - Did the staff use any resources to explain you the process?
        - Probe – what was used and was it useful?
        - Probe – what would have been helpful?
    - Can you tell me a bit about any other resources that you have seen, are aware of or used that you have found useful in these situations:
        - To access end-of-life care
        - To make decisions about end-of-life care
        - To help you with communicating about end-of-life care with provider/staff/health service

3.Environmental context and social Influences

- Can you tell me what does your wider community think about EOLC planning?
- In your view, is cancer care system able recognise and respond to this perspective while planning EOLC?
- How about responding to multiple perspectives across different CALD communities?
    - If positive, probe what has been effective/good/useful

- What are the specific challenges?
- Probe differences for their particular community
- What might be the solutions for improving EOLC planning for CALD communities?

4. If EOLC planning was conducted to the best possible quality in cancer care, what would that look like?
   a. What needs to happen to get the system there?

5. Is there anything else you would like to add?
