# Supplementary material for: Supporting Advance Care Planning Among Mandarin and Cantonese Speaking Communities: A Qualitative Exploratory Study
Source: Curr Oncol. 2026 May 14;33(5):288. doi: 10.3390/curroncol33050288 (PMC13204915; doi:10.3390/curroncol33050288)
Supplement: Supplementary file 1 [file curroncol-33-00288-s001.zip › SuppFile_03_IG__CARERs_V_15_02_2024.pdf]

<u>Interview Guide:2</u>

1.Introductions & Carer Duties

- Could you please tell me a bit about your background.
- Could you please tell me who you are caring/cared for, how long and which cancer services you have attended?
- Can you list your duties as a carer ?
- Are/were you the primary person taking care of communication during this time?
- If no – Who was? Can you describe the process? Did you have any role?
- If yes, were you involved in any discussion or communication about EOL planning in your carer role?

Probes for Process: was information being provided to you only or in a family-meeting? how were you passing the info to pt? was pt. okay to receive all the information?

2. Knowledge, skills, capabilities and resources

- Can you tell me a bit about your experiences of communication with health professionals about EOLC planning – wishes, preferences or questions you might have had?

    - Probe – how did these conversations come about at what stage and in what setting
    - Probe – what made it easier for you and your loved ones to talk about your wishes preferences and questions
    - Probe – what challenges did you encounter in having these conversations? did any of these challenges relate to your cultural or linguistic background?
    - From your experiences above, do you think cancer service staff approaches EOLP in a culturally sensitive manner?

    - Did the staff use any resources to explain you the process?
        - Probe – what was used and was it useful?
        - Probe – what would have been helpful?
    - Can you tell me a bit about any other resources that you have seen, are aware of or used that you have found useful in these situations:
        - To access end-of-life care
        - To make decisions about end-of-life care
        - To help you with communicating about end-of-life care with provider/staff/health service

3. What changes can be made in the system to make it work better for ethnic groups like yourself?
